# A Real-Time Recombinase Polymerase Amplification Assay for Specific Detection of Lumpy Skin Disease Virus

**DOI:** 10.3390/vetsci10100625

**Published:** 2023-10-19

**Authors:** Qi Zhai, Xia Zhou, Liyin Du, Nan Yang, Yakun Lou, Jianying Liu, Shaolun Zhai

**Affiliations:** 1 Institute of Animal Health, Guangdong Academy of Agricultural Sciences, Key Laboratory of Livestock Disease Prevention of Guangdong Province, Scientific Observation and Experiment Station of Veterinary Drugs and Diagnostic Techniques of Guangdong Province, Ministry of Agriculture and Rural Affairs, Guangzhou 510640, China; zhaiqi@gdaas.cn (Q.Z.); zhouxia@gdaas.cn (X.Z.); 2 Zijin Animal Disease Prevention and Control Center, Zijin 517400, China; gdshysdly@sina.com; 3 Zhengzhou Zhongdao Biotechnology Co., Ltd., Zhengzhou 451000, China; 18736090876@163.com; 4 Guangdong Agricultural Technology Extension Center, Guangzhou 510520, China

**Keywords:** lumpy skin disease, lumpy skin disease virus, real-time recombinase polymerase amplification, specific detection, bovine

## Abstract

**Simple Summary:**

Lumpy skin disease virus (LSDV) poses a significant threat to the livestock industry, causing considerable economic losses due to decreased animal productivity, hide quality, and reproductive efficiency. In China, the goat pox vaccine (AV41 strain) has been used to combat LSDV; previous detection methods could not easily differentiate between LSDV and the vaccine strain. The real-time recombinase polymerase amplification (RPA) method developed in this study offers a rapid, specific, and sensitive solution for detecting LSDV, reducing diagnostic time, and minimizing the risk of false positives. The high consistency of this method with the real-time PCR recommended by the World Organisation for Animal Health (WOAH) further highlights its potential for widespread application in clinical diagnosis and LSDV detection in China. By enabling more accurate and efficient diagnosis, this novel technique can help improve disease prevention and control strategies, ultimately benefiting the livestock industry and reducing economic losses.

**Abstract:**

Lumpy skin disease virus (LSDV) infection, accompanied by loss of hide quality, poor reproductive efficiency, consistent degenerative emaciation, and milk yield reduction of animals, causes severe economic implications in endemic zones. The heterologous attenuated goat pox (GTPV) vaccine (AV41 strain) was used in China to prevent LSDV infection. Only a few LSDV detection methods that distinguish LSDV from GTPV vaccine strains have been reported before. For simple, rapid, and specific detection of LSDV, the real-time recombinase polymerase amplification (RPA) method was established with the specific primers and probes designed according to the conserved regions of ORF132 gene sequences. The assay could be finished within 20 min at a constant temperature (39 °C). This method had a limit of detection (LOD) of 15 copies/μL for LSDV and no cross-reaction with the nucleic acids of goat pox virus, infectious bovine rhinotracheitis virus, Pasteurella multocida, and bovine healthy tissue. Furthermore, 43 clinical samples were detected by this method and the real-time PCR recommended by the World Organisation for Animal Health (WOAH), with a kappa value, was 0.94. These results demonstrated that the real-time RPA method for detecting LSDV developed in this study was characterized by high sensitivity and specificity, which has wide application value in the clinical diagnosis and detection of LSDV in China.

## 1. Introduction

Lumpy skin disease (LSD) is a contagious viral disease caused by the causative agent lumpy skin disease virus (LSDV) with a double-stranded DNA genome of approximately 151 kb. LSDV, which belongs to the Capripoxvirus genus within the Poxviridae family, is closely related to the other Capripoxvirus, goat pox virus (GTPV) and sheep pox virus (SPPV) [1]. The three viruses have several subtle genetic variations, causing differences in the pathogenesis and host range of the capripoxviruses, especially the presence of LSDV and GTPV-specific gene ORF132 [2,3].

LSDV can infect cattle and buffalos naturally, displaying pyrexia, generalized pox lesions of the skin and internal organs, and generalized lymphadenopathy. Additionally, it causes a significant reduction in milk yield in cows and can induce temporary or permanent infertility in bulls [4,5]. Historically, LSD was first reported in Africa [6]. However, due to economic development and increased trade, its geographic distribution has expanded dramatically, recently affecting the Middle East, the European continent, and Central Asia [7,8]. More recently, LSD has started spreading into East Asia and South Asia, like China [9], India [10], Bangladesh [11], Thailand [12], and Vietnam [13]. The spreading of LSD caused substantial economic losses in the local cattle industry [14,15].

At present, the effective strategy for LSDV eradication and control is vaccination, which can utilize either heterologous vaccine preparations, typically based on sheep pox or goat pox viruses, or homologous LSDV vaccines, such as the Neethling vaccine and KSGP O240 [16]. For biosafety reasons, the Ministry of Agriculture and Rural Affairs of the People’s Republic of China only recommends vaccinating susceptible animals with a heterologous attenuated goat pox AV41 strain vaccine. In most cases, using the live attenuated vaccine to prevent LSD is effective [17]. However, the live attenuated vaccine could also replicate in vaccinated animals and cause adverse reactions in some clinical cases [18,19]. Therefore, developing an effective specific surveillance tool is necessary to differentiate LSDV from the GTPV vaccine strain.

Recombinase polymerase amplification (RPA) is an isothermal amplification technique that relies on three essential enzymes: recombinase (for primer-template DNA pairing), DNA polymerase (for amplification and extension), and single-stranded DNA-binding protein (for stabilizing the DNA configurations). The RPA system is conducted without the need for a complex thermal cycler and can be completed within 30 min at a constant temperature (37–42 °C) using a simple water bath. The inclusion of a specific fluorescent probe in the RPA system enables real-time monitoring, referred to as real-time RPA [20].

In this study, a real-time RPA assay for the detection of lumpy skin disease virus was established and compared for consistency with the real-time PCR recommended by the World Organisation for Animal Health (WOAH). This method can be more convenient for LSDV detection without affecting the accuracy of the test results, especially for some farms with simple test conditions. More importantly, it is also significant for the availability of diagnostic assays capable of specific detection for LSDV isolates but not GTPV vaccine strains.

## 2. Materials and Methods

### 2.1. Strains of Virus, Bacteria Strains, and Clinical Samples

Goat pox virus (GTPV, AV41 strain), Infectious bovine rhinotracheitis virus (IBRV, LY strain), and *Pasteurella multocida* (HY strain) were preserved in our laboratory. A total of 32 positive clinical samples of LSD, including EDTA blood, nasal and oral swabs, and skin nodule tissue, were supplied by the animal disease prevention and control center of Guangdong Province. Our laboratory obtained 11 negative clinical samples and healthy bovine tissue from different farms and stored them at −20 °C until use.

### 2.2. DNA Extraction

The DNA of the virus from clinical samples was extracted using the QIAamp DNA mini kit (Qiagen, Dusseldorf, Germany) according to the manufacturer’s instructions and was stored at −80 °C until needed.

### 2.3. Primers and Probes Design

The LSDV ORF132 sequence (MH646674, position 119,870–120,400) was obtained from GenBank and aligned with the GTPV AV41 vaccine strain using the multiple sequence alignment tool DNAMAN (the versions 7.0.2.176). After alignment and analysis, a specific primer–probe set (Table 1) that can differentiate the GTPV AV41vaccine and LSDV strains was designed according to the TwistAmp exo kit manual (TwistDX, Cambridge, UK). Meanwhile, Primer-blast in NCBI was run to check the specificity of the primers and probes. Then, the primers and probes were synthesized by Sangon Biotech Co., Ltd. (Shanghai, China).

### 2.4. Generation of Standard DNA

The ORF132 gene fragment of LSDV was incorporated into the recombinant plasmid pUC57-ORF132, which was synthesized by Sangon Biotech Co., Ltd. DNA concentration was quantified using Thermo Scientific NanoDrop Lite (Wilmington, DE, USA), and the copy number was calculated as the following equation:

$$Number\;of\;copies\;per\;µL=\frac{c\times 6.022\times 10^{23}}{n\times 660}$$

where *c* = concentration of pUC57-ORF132 (g/µL); *n* = number of base pairs in a single pUC57-ORF132.

### 2.5. Real-Time RPA Assay

The RPA assay was performed in a 50 µL volume recommended by the TwistAmpTM exo lyophilized kit (TwistDX, Cambridge, UK). The reaction system included 29.5 μL of rehydration buffer, 1.5 μL of forward and reverse primer (10 µM), a 1 μL probe (10 µM), a 5 μL DNA template, 2.5 μL of magnesium acetate (280 mM), and 9 μL of nuclease-free water. A reaction mixture using ultrapure water instead of a DNA template served as a negative control. The RPA assay was performed in an MA-1600 isothermal fluorescence PCR system (Molarray, Suzhou, China) at 39 °C for 20 min in the 6-carboxy-fluorescein (FAM) channel. The threshold time (TT) was calculated based on the “fluorescence increase above threshold” when the RPA reaction was completed.

### 2.6. Analytical Sensitivity and Specificity Test

To determine the sensitivity of the RPA assay, the pUC57-ORF132 plasmid was diluted in a 10-fold gradient with nuclease-free water to 10^3^ copies/μL, 10^2^ copies/μL, 10 copies/μL, and 1 copy/μL. The assay was carried out three times. The estimated detection limit is determined when all replicates in each dilution step (TT value presents) are detected. Then, several gradient concentration plasmids were prepared near the estimated detection limit, and each concentration was repeatedly detected 20 times. The lowest detection limit was the concentration level, with a positive detection rate of more than 95%.

The nucleic acid of GTPV, IBRV, *Pasteurella multocida*, and healthy bovine tissue was tested to evaluate the assay’s specificity, and the positive and negative controls were also simultaneously run.

### 2.7. Validation of Performance Using Clinical Samples

The performance of the RPA assay was evaluated with 43 clinical samples. For comparison, all the samples were tested with the real-time PCR method recommended by the WOAH in parallel [21]. The RPA assay’s diagnostic sensitivity and specificity were evaluated with the WOAH real-time PCR as the standard. The kappa value and *p*-value were calculated using SPSS for Windows version 22 (SPSS, Chicago, IL, USA), as reported previously [22,23], and *p*-value (*p*) < 0.05 was considered as statistically significant.

## 3. Results

### 3.1. Primer and Probe Selection

**Figure 1:**
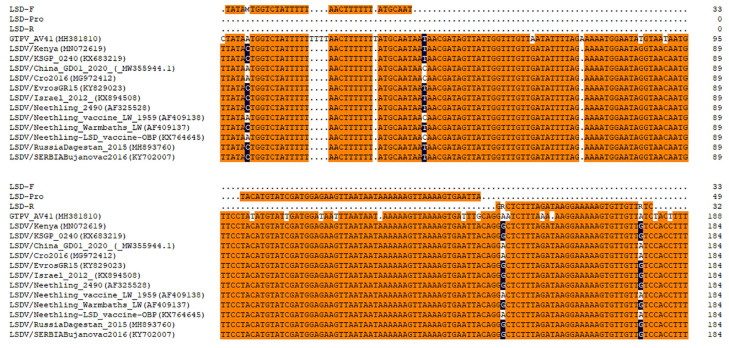
Multiple sequence alignment of the real-time RPA assay sequences with GTPV AV41 vaccine strain and different LSDV isolates.

Through the multiple sequences alignment and analysis, the selected forward primer sequence (LSD-F) has a difference of 5 bases from the GTPV AV41 vaccine strain, including a 4-nt deletion (TTTT). The reverse primer (LSD-R) and the probe (LSD-Pro) sequence have differences of 3 bases and 7 bases from the vaccine strain, respectively (Figure 1).

### 3.2. Analytical Sensitivity and Specificity Analysis

**Figure 2:**
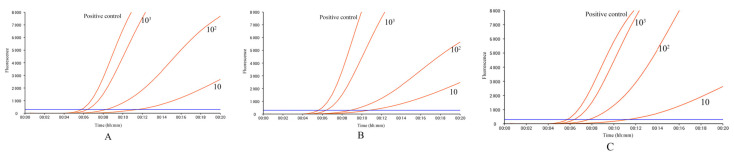
Analytical sensitivity analysis of the real-time RPA assay. 10-fold gradient dilutions of pUC57-ORF132 plasmid ranging from 10^3^ to 10^0^ copies/μL were used as the template for estimating the detection limit. The (**A**–**C**) represents three replications.

For the analytical sensitivity test, serial dilutions of pUC57-ORF132 plasmid were used to determine the LOD. The results showed that the detection rate of 10^3^ copies/μL, 10^2^ copies/μL, and 10 copies/μL was 100%, and 1 copy/μL was not detected in the three duplicate assays (Figure 2), so 10 copies/μL was taken as the estimated detection limit.

Samples with gradient concentrations of 15 copies/μL, 10 copies/μL, 5 copies/μL, and 1 copy/μL were prepared near the estimated detection limit, and each concentration was repeated 20 times. The results showed that the detection rates of samples with concentrations of 15 copies/μL and 10 copies/μL were 100% and 90%, respectively. The detection rate of 5 copies/μL is 50%, and 1 copy/μL is not detected (Table 2), so the 15 copies/μL is determined as the LOD.

**Figure 3:**
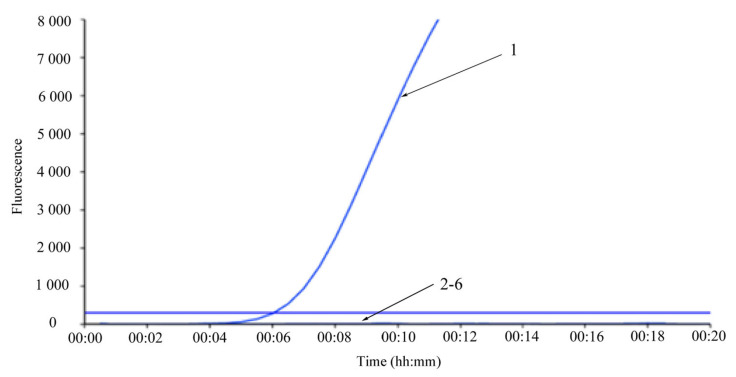
Analytical specificity analysis of the real-time RPA assay. 1: Positive templates of LSDV. 2–6: GTPV, IBRV, Pasteurella multocida, healthy bovine tissue, and nuclease-free water control.

As for the analytical specificity test, a specific fluorescence signal was observed only from the corresponding LSDV nucleic acid. None of the other selected viruses and bacteria strains tested positive, demonstrating the superior specificity of the RPA assay (Figure 3).

### 3.3. Qualitative Agreement Test between the Real-Time RPA Assay and WOAH Real-Time PCR Method

Forty-three samples were evaluated by both the real-time RPA assays and the WOAH real-time PCR method. Among these clinical samples, the RPA assays detected 31 positive and 12 negative samples, while the WOAH real-time PCR detected 32 positive and 11 negative samples. Taking the WOAH real-time PCR as the standard, the diagnostic sensitivity and specificity of the real-time RPA assay were estimated to be 96.88% and 100% (Table 3), respectively. The kappa value was 0.94, exhibiting a strong agreement between the two methods, and the *p*-value was 6.4349 × 10^−10^, indicating strong statistical significance. The results suggested the potentiality of the RPA assay in clinical diagnosis.

## 4. Discussion

LSDV is a poxviral threat pathogen of cattle currently widespread in Asia, and the disease situation is not encouraging [24]. The availability of diagnostic assays capable of differentiating detection among LSDV, SPPV, and GTPV, especially the differentiation between field LSDV isolates and vaccine strains, is urgently needed.

Recently, new diagnostic methods for detecting LSDV have been developed. A real-time RPA assay for detecting the LSDV genome, targeting GPCR genes, was established in 2016 [25]. An innovative diagnostic method targeting the LSDV-ORF068 gene, employing an RPA-CRISPR-Cas12a integration (RPA-Cas12a-fluorescence assay), exhibited a high sensitivity with 5 copies/μL without any cross-reactivity to prevalent bovine viruses [26]. However, these RPA assays can simultaneously detect SPPV and GTPV, and do not apply to the diagnosis and surveillance of LSDV in China due to the heavy use of the GTPV AV41 strain vaccine in cattle farms. A real-time HRM PCR assay based on a high-resolution melting curve analysis for detection and differentiation between field isolates and vaccine strains of LSDV was developed in 2018 [27]. Researchers have developed several real-time quantitative RT-PCR assays for detecting and differentiating field-type and vaccine strains of LSDV [28,29]. The two detection methods can distinguish LSDV wild strains from vaccine strains but require expensive and high-precision qPCR instruments with complex reaction procedures. It has application value during vaccination based on homologous LSDV vaccine strains but is also unsuitable for China.

The TaqMan assay systems for differentiation between LSDV and GTPV were developed recently [30,31]. Compared to probe-based real-time PCR, the real-time RPA assay requires simpler and less-expensive qPCR instruments and can save reaction time and accelerate detection progress. Therefore, for farms and laboratories without a sophisticated thermal cycler or with limited funds, real-time RPA is a practical and cost-effective option. In this study, we described the development of a new real-time RPA assay for the specific detection of LSDV. The design of the primers and probes was based on the ORF132 gene sequences of LSDV, which have some differences and deletions compared to the GTPV AV41 sequence, and this design can enable specific detection of LSDV isolates, including the recombinant LSDV circulating in China. The RPA with the fluorescent probe method amplifies DNA rapidly at a constant temperature (39 °C), and the detection can be finished within 20 min, which is simple and time-saving. This method was highly sensitive with the minimum detection limit of 15 copies/μL for LSDV, meanwhile there was no cross-reaction with GTPV, IBRV, *Pasteurella multocida*, and healthy bovine tissue. Forty-three clinical samples were detected by this method and the WOAH real-time PCR with good consistency. However, there are no commercially available and recognized LSDV antibody test kits in China; we cannot conduct a comparison with antibody testing methods. The results demonstrated that this study’s real-time RPA detection method merits in specific detection of LSDV DNA, high sensitivity, convenient operation, and short detection time, which is suitable for LSDV surveillance and diagnostic need in China.

## 5. Conclusions

In summary, a real-time RPA amplification assay for specific detection of lumpy skin disease virus was established, which has wide application value in clinical diagnosis and helps us better control LSDV in China.

## Figures and Tables

**Table 1:** Primers sequence and probe for the RPA assay.

Name	Sequence 5′–3′	Amplicon Length (bp)
LSD-F	TATAMTGGTCTATTTTTAACTTTTTTATGCAAT	176
LSD-R	GAYAACAACACTTTTTCCTTATCTAAAGAGYC	
LSD-Pro	TAATTCACTTTTAACTTTTTTATTAT/i6FAMdT/A/idSp/A/iBHQ1dT/CCATCGATACATGTA-C3 Spacer	

**Table 2:** Minimum detection limit test results.

Reaction Times	15 Copies/μL		10 Copies/μL		5 Copies/μL		1 Copy/μL	
	TT Value (min)	Result	TT Value (min)	Result	TT Value (min)	Result	TT Value (min)	Result
1	00:11:04	+	00:14:42	+	00:17:44	+	Unde	-
2	00:10:16	+	00:14:46	+	00:17:58	+	Unde	-
3	00:10:31	+	00:17:19	+	00:18:13	+	Unde	-
4	00:13:16	+	00:14:26	+	00:16:38	+	Unde	-
5	00:09:51	+	00:15:21	+	00:15:38	+	Unde	-
6	00:09:47	+	00:14:52	+	Unde	-	Unde	-
7	00:10:37	+	00:15:35	+	00:15:56	+	Unde	-
8	00:08:43	+	00:13:37	+	Unde	-	Unde	-
9	00:09:27	+	00:11:25	+	Unde	-	Unde	-
10	00:08:47	+	00:14:24	+	Unde	-	Unde	-
11	00:08:25	+	00:12:44	+	Unde	-	Unde	-
12	00:11:27	+	Unde	-	Unde	-	Unde	-
13	00:08:51	+	00:14:40	+	00:18:01	+	Unde	-
14	00:10:05	+	00:12:28	+	00:17:12	+	Unde	-
15	00:08:57	+	00:14:00	+	00:17:34	+	Unde	-
16	00:09:17	+	Unde	-	00:16:33	+	Unde	-
17	00:08:18	+	00:09:04	+	Unde	-	Unde	-
18	00:08:17	+	00:14:28	+	Unde	-	Unde	-
19	00:09:03	+	00:12:02	+	Unde	-	Unde	-
20	00:10:44	+	00:13:48	+	Unde	-	Unde	-

Note: “Unde” means that no signal is detected, “+” means that the result is positive, and “-” means that the result is negative.

**Table 3:** The detection results of 43 clinical samples using the real-time RPA assay and the WOAH method.

		Real-Time RPA			Diagnostic Sensitivity	Diagnostic Specificity	Kappa Value
		Positive	Negative	Total			
WOAH method	Positive	31	1	32	96.88%	100%	0.94
	Negative	0	11	11			
	Total	31	12	43			

Note: Diagnostic sensitivity = no. of true positives/(no. of true positive + no. of false negatives), and diagnostic specificity = no. of true negatives/(no. of false positive + no. of true negatives).

## Data Availability

The authors confirm that the data supporting the findings of this study are available within the article.
